# Advances in Brain Tumor Surgery for Glioblastoma in Adults

**DOI:** 10.3390/brainsci7120166

**Published:** 2017-12-20

**Authors:** Montserrat Lara-Velazquez, Rawan Al-Kharboosh, Stephanie Jeanneret, Carla Vazquez-Ramos, Deependra Mahato, Daryoush Tavanaiepour, Gazanfar Rahmathulla, Alfredo Quinones-Hinojosa

**Affiliations:** 1 Department of Neurosurgery, Mayo Clinic Florida, 4500 San Pablo Road, Jacksonville, FL 32224, USA; lara-velazquez.montserrat@mayo.edu (M.L.-V.); Alkharboosh.Rawan@mayo.edu (R.A.-K.); stjeanner@utexas.edu (S.J.); Ramos.Carla@mayo.edu (C.V.-R.); Mahato.Deependra@mayo.edu (D.M.); 2 Department of Medicine, National Autonomous University of Mexico (UNAM), Av. Universidad, Coyoacan, Mexico City 04510, Mexico; 3 Mayo Clinic College of Medicine, Mayo Clinic Graduate School, Rochester, MN 55905, USA; 4 Department of Psychology, The University of Texas at Austin, 108 E Dean Keeton St, Austin, TX 78712, USA; 5 Department of Neurosurgery, University of Florida College of Medicine, 653 8th St W., Jacksonville, FL 32209, USA; Daryoush.Tavanaiepour@jax.ufl.edu

**Keywords:** brain tumor surgery, laser therapy, awake craniotomy, novel treatments for glioma, optical coherence tomography

## Abstract

Glioblastoma (GBM) is the most common primary intracranial neoplasia, and is characterized by its extremely poor prognosis. Despite maximum surgery, chemotherapy, and radiation, the histological heterogeneity of GBM makes total eradication impossible, due to residual cancer cells invading the parenchyma, which is not otherwise seen in radiographic images. Even with gross total resection, the heterogeneity and the dormant nature of brain tumor initiating cells allow for therapeutic evasion, contributing to its recurrence and malignant progression, and severely impacting survival. Visual delimitation of the tumor’s margins with common surgical techniques is a challenge faced by many surgeons. In an attempt to achieve optimal safe resection, advances in approaches allowing intraoperative analysis of cancer and non-cancer tissue have been developed and applied in humans resulting in improved outcomes. In addition, functional paradigms based on stimulation techniques to map the brain’s electrical activity have optimized glioma resection in eloquent areas such as the Broca’s, Wernike’s and perirolandic areas. In this review, we will elaborate on the current standard therapy for newly diagnosed and recurrent glioblastoma with a focus on surgical approaches. We will describe current technologies used for glioma resection, such as awake craniotomy, fluorescence guided surgery, laser interstitial thermal therapy and intraoperative mass spectrometry. Additionally, we will describe a newly developed tool that has shown promising results in preclinical experiments for brain cancer: optical coherence tomography.

## 1. Introduction

Glioblastoma (GBM), a grade IV glioma, according to World Health Organization (WHO) classification, is the most lethal primary glioma in adults. Several studies have shown that patients with GBM have poor survival [1,2,3]. GBM has a prevalence of 26,000 cases, with a mortality rate of 15,000 cases yearly in the US, and an incidence of two to three per 100,000 adults per year. GBM accounts for 52% of all primary brain tumors in the US, as stated by the National Cancer Institute [4]. Standard therapy, including surgery along with chemotherapy and radiation, has insignificantly improved GBM patient’s survival. Newly diagnosed GBM patients with favorable Karnofsky performance scale (>70%) (KPS) and who undergo the standard of care including surgical resection, chemotherapy and radiation, have a survival mean of approximately 15 months, with only 10% of patients living more than 5 years [5]. Furthermore, the mean survival for those patients who undergo surgical resection alone is significantly longer than those who have only undergone biopsy, 7 versus 3.5 months, respectively [6]. Hence, the main prognostic factor for these patients’ survival with GBM is the extent of resection of the tumor [3,7,8,9]. Furthermore, the infiltrative capacity of GBM cells makes complete eradication of cancer cells impossible to achieve. Due to the pattern of glioma cell infiltration, their migratory capacity allows them to collect below the pial margins, surround the vasculature, and migrate through white matter tracts. Their diffusive nature over long distances allows for a more aggressive disease, making recurrence 100% [10]. Among the histological characteristics that contribute to GBM propagation are the high proliferative rate, necrosis, angiogenesis, and the existence of a specific subgroup of cells named Brain Tumor Initiating Cells (BTIC), which have the ability to survive even the most eradicative treatments [11]. BTICs have been associated to tumor development, and are responsible for tumor recurrence secondary to their self-renewing ability. Their migratory capacity allow them to cross the corpus callosum where they could be found at least 3 cm away from their primary site [12]. In addition, they can maintain an undifferentiated state due to the expression of several transcription factors (e.g., Oct4, Sox1, Wnt/β catenin, SOX2 and STAT3) commonly present in normal somatic or embryonic cells, which are fundamental for cellular stemness [13,14]. Genetic mutations in tumor suppressor genes such as TP53, Rb, and deletion of chromosome 1p19q are contributors for tumor resistance [15]. The genetic signature exhibited by each tumor, allowed its classification into neural, proneural, mesenchymal and classical molecular subtypes. The neural subtype commonly expresses markers such as NEFL, GABRA1, SYT1 and SLC12A5. The proneural subtype is associated with PDGFRA abnormalities and IDH1 and TP53 mutations. The classical subtype is related with EGFR mutations; while high expression of CHI3L1 and mutations in MET and NF1 genes are common findings in the mesenchymal class. Interestingly, patients with classical and mesenchymal subtypes exhibit better therapy response to chemo- and radiotherapy or repeated cycles of chemotherapy than those with the proneural genotype [16,17]. Furthermore, earlier studies suggested a shift in the molecular subtype of the primary mass after recurrence, most likely towards a mesenchymal subtype [18]. However, a mutual agreement among researchers has not been reached.

Among many therapeutic strategies against GBM, surgical excision and extent of resection are essential components for determining both tumor diagnosis and patient prognosis, all of which depend on the surgeon’s capability to discriminate between cancer and non-cancer tissues. Therefore, techniques that facilitate a well-demarcated differentiation between normal versus cancer-invaded parenchyma are essential in order to achieve a maximal surgical excision.

In this review, we will briefly describe the current standard of care for newly diagnosed GBM, as well as strategies for recurrent GBM. We will also describe several techniques that have improved glioma surgery through a physiological delineation enhancement of the tumor (cortical mapping for awake craniotomies), through tumor anatomy delineation (fluorescence-guided surgery and laser interstitial thermal therapy) or through metabolite depiction of the samples (intraoperative mass spectrometry). The ability to diagnose tumors intraoperatively has considerably advanced the field of brain tumor surgery; the review will specifically take account of widely used practical technologies that have enabled reliability in identifying lesions at the tumor-brain interface such as (i) Confocal Intraoperative Microscopy, which can facilitate spatial selection of highly heterogeneous tissue for molecular and tissue diagnosis; (ii) Fluorescence Guided Surgery, which allows the selective uptake of a compound by tumor cells mitigating the difficulties associated with a brain tumors infiltrative nature; (iii) Laser Interstitial Therapy, which allows the treatment of tumor in patients who do not respond to stereotactic radiosurgery or have radiation-treatment associated necrosis; and (iv) Intraoperative Mass Spectrometry, which provides another avenue of extent of tumor resection and immediate feedback, since biopsies are often the only source of intraoperative diagnostic information leaving much predictive, prognostic, and diagnostic information undiscovered. Finally, we will discuss optical coherence tomography, an evolving technology in preclinical stages, which has shown promising results for intraoperative identification of cancer samples that will enhance tumor resection.

Although many more techniques exist within the realm of intraoperative therapy, such as Intraoperative Focused Ultrasound and Computed Tomography, these techniques require a more comprehensive review considering the limitations of the technique in identifying the entirety of glioma in the absence of a suitable adjuvant. Furthermore, contrast-enhanced areas in such highly invasive disease pathology is limited to cases in specific areas. Such drawbacks are corrected by photodynamic diagnostics such as 5-ALA and others, which will be carefully reviewed in this paper.

## 2. Newly Diagnosed and Recurrent GBM: Treatment & Management

De novo GBMs occur in 60% of lesions, whereas secondary GBMs (a progression from low-grade to high-grade lesions) occur in 40% [19,20]. Several factors are attributed to GBM prognosis, depending on clinical and biological patient parameters (e.g., age and KPS), or based on the characteristics of the tumor (e.g., Ki67-mitotic index, necrosis, vascular proliferation, location-proximity to eloquent areas or the sub ventricular zone, and genetic alterations, such as IDH mutation and MGMT methylation) [19]. The current standard therapy for newly diagnosed GBM consists of maximal tumor resection plus daily administration of temozolamide (TMZ) at a dose of 75 mg/m^2^ and 60 Gy of radiotherapy (2 Gy daily) for 4–6 weeks, followed by a TMZ maintenance dose of 150–200 mg/m^2^ for five days every 28 days for six cycles [5]. This treatment paradigm has improved the median survival from 12.1 months to 14.6 months [15]. Still, at least 50% of GBM patients are resistant to TMZ treatment due to either an overexpression of methylguanin-DNA-methyltransferase (MGMT), which is responsible for initiating DNA repair against alkylating chemotherapeutic agents such as TMZ, or a methylation of the promoter site stifling protein expression [21]. Interestingly, an extended administration of the TMZ scheme for 12 cycles has led to an improvement in progression-free survival (PFS) from 12.8 to 16.8 months [22]. However, despite multimodal approaches, the recurrence rate in GBM is almost 100% [23].

GBM tends to infiltrate normal parenchyma through diverse growth patterns and, although less common, it may spread in the ventricles, neither of which emit any growth signals [24]. Due to this infiltrative nature, the remaining cancer cells in the post-surgical cavity can form a new mass within 2–3 cm from the border of the original lesion [24]. Additionally, the resilient nature of these cells to previous treatments displays a worse panorama, exhibiting a survival period between 3 to 9 months (progression-free survival (PFS) of 6 months in 25% of the cases) and a treatment-response rate of only 5 to 10% [25].

Currently, there are no universal treatments for patients with recurrent GBM. Strategies for facing this situation vary among venues, where present options include repeated resections with probed prolonged survival, as demonstrated in patients subjected to a second, third, or fourth intervention, who show an improved survival of 15.4, 22.4, and 26.6 months, respectively [26], as well as systemic application of a second or third line of cytotoxic treatments (TMZ or bevacizumab (BVZ) respectively), modified irradiation schemes, radiosurgery, and more recently, tumor treatment fields [27,28]. The first-line pharmacological option is BVZ, a monoclonal antibody against vascular endothelial growth factor (VEGF) FDA approved [29], that has shown benefits in combination with cytotoxic agents such as irinotecan, as demonstrated in clinical trials developed by Vredenburgh et al., 2007 and Firedman et al., 2009 [30,31]. Another current strategy for recurrent GBM is the local intraoperative chemotherapeutic agent carmustine (BCNU) [32,33,34,35,36]. Despite its local application directly into the tumor bed and some improved survival results, BCNU has been shown in some studies to have a 43% complication rate, in addition to the high cost of the treatment ($7800, in addition to $20,000 for surgery and radiation), as reported in some studies [18].

Additional agents used against GBM include other nitrosoureas such as fotemustine, which can be administered as a single agent or in combination with PCV (P: procarbazine, C: lomustine and V: vincristine). Carboplatin, etoposide and irinotecan are other regimens that have shown modest efficacy after single or combined administration [37]. Clinical trials with cediranib, gefitinib and erlotinib for recurrent GBM have not shown an improvement in prolonging survival so far [38,39,40].

Thus, chemotherapy, either for GBM of recent diagnosis or for a recurrent mass, is not a resolutive solution. GBM cells’ ability to escape to cytotoxic agents through different venues (overexpression of proteins related to cellular cycle and angiogenesis, as well as drug-excision pumps) prompt surgeons to eliminate as much cancer tissue as possible, without compromising patient stability, during surgery [41].

The TTF (Tumor Treating Fields) device is a novel concept for treating GBM. This technology is based on the delivery of low- to intermediate-frequency electrical fields that selectively kill proliferating cells when placed on the scalp [42]. The effect is mediated through the disruption of mitotic spindle formation during mitosis. TTF prolonged the median PFS survival (3 months approximately) when used in combination with the adjuvant temozolamide [43].

## 3. Surgery for Glioblastoma: Advances and Challenges

Despite the available adjuvant options for GBM, survival rates have not dramatically changed, compared with other types of cancers, such as breast cancer, according to the Centers for Disease Control and Prevention, (CDC) [44].

Hence, surgery is an important modality for establishing diagnosis (through histopathological confirmation after tissue examination), and improving prognosis while maintaining patient pre-intervention functional activity, since the extent of resection is the main decisive factor for survival [1,45,46]. Indeed, surgery serves as a curative means for the disease, as evident through the treatment of low grade gliomas [47].

Gross total resection (GTR) of high grade gliomas (HGG) and low grade gliomas (LGG) increases the median survival rate by 200% and 160% respectively, when compared to survival rates for patients subjected to a subtotal resection (STR) [7,48]. In a retrospective systematic meta-analysis study performed on over 41,000 newly diagnosed GBM patients, it was found that GTR proved superior over STR with a 61% increase in likelihood of a one-year survival and a 51% likelihood of a 12-month progression free survival [49].

**Figure 1:**
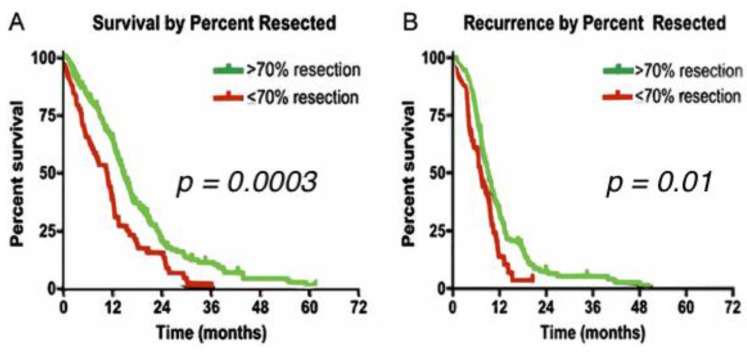
Kaplan Meyer curves of extent of resection in high grade gliomas (HGG). (**A**) The median survival for patients with >70% tumor resection was 14.4 months compared with 10.5 months for patients with ≤70% resection (*p* = 0.0003); (**B**) The median percent free survival for patients with >70% tumor resection was 9.0 months compared with 7.1 months for patients with ≤70% resection (*p* = 0.01). (Reproduced from Chaichana et al. [7]).

Although a complete eradication of HGG due to the microscopic infiltrative cells is an unachievable task, a 90% threshold of resection without compromising functional pathways remains as the realistic desired goal of every neurosurgeon. In fact, even STR of 70% has shown statistically significant improvement in overall survival and seizure control [7]. When maximal resection is not feasible, supramarginal resection (SMR) of the tumor is an option, which is defined as doing resection beyond tumor mass enhancement displayed by the imaging techniques (Figure 1).

Throughout the years, innovations in neurosurgical oncology have led to the possibility of obtaining a maximal cytoreduction while preserving functional pathways [50]. Radiographic analysis such as Intraoperative magnetic resonance imaging (iMRI) for defining the tumor’s location, edema, and involvement of eloquent areas are crucial tools to determine the appropriate surgical intervention in every patient. Furthermore, imaging has also a placed in determining the prognosis in patients such as cases with a butterfly glioblastoma (a tumor involving both hemispheres via invasion of the corpus callosum) [8], where poor prognosis is expected as demonstrated by Chaichana et al., who showed significant decreased overall survival of butterfly GBM of 7.0 months versus non-butterfly GBM of 11.6 months that were matched for age, KPS tumor size, extent of resection (EoR), and post-op adjuvant therapy [8].

Thus, iMRI has become an essential tool that involves the obtainment of real-time images of the patient’s brain during the surgical procedure, enabling the surgeons to evaluate if a complete resection was achieved or further resection is needed before closing the surgical field [9]. This tool also decreases the risk of damaging principal areas in the brain during surgery, allowing an extent of resection of 99.78% in gliomas adjacent to eloquent areas [51]. Despite the probed benefits exhibited by this tool, high cost (variable among institutions) and time consumption (30–40 min approximately) are strong disadvantages to consider [51,52].

Techniques such as cortical mapping of the brain, fluorescence-guided surgery, laser interstitial thermal therapy and intraoperative mass spectrometry are used nowadays in the operating room for tumor resection. In the near future, evolving technologies such as an optical coherence tomography will revolutionize the surgical field of central nervous system(CNS) gliomas, allowing real-time tumor delineation in a short period of time.

## 4. Current Trends in Glioblastoma Surgery

**Figure 2:**
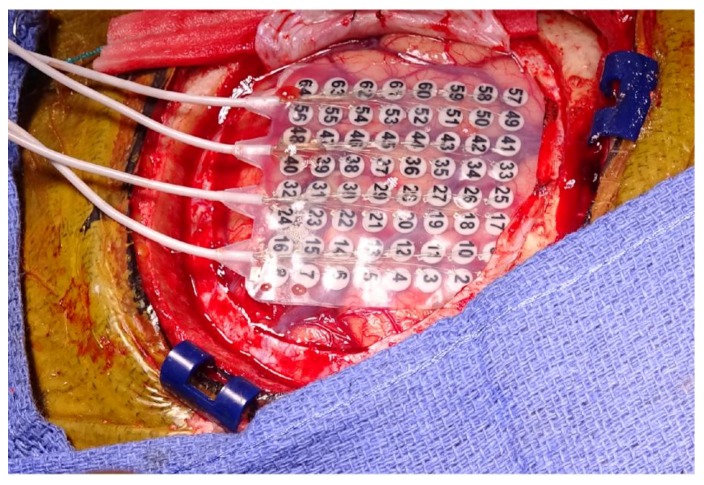
Awake craniotomy for cortical mapping and tumor resection. Electrode placement for monitoring and localization of eloquent areas. (Original image).

Awake Craniotomy: Awake craniotomy (AC) is a useful anesthetic and surgical technique that allows for the identification of eloquent areas in tumors involving cortical and subcortical regions, especially for tumors that would otherwise be considered inoperable [47,53,54,55,56,57]. There are cases during repeat awake craniotomy where the tumor can displace the eloquent areas due to brain plasticity; thus, the surgeon cannot rely on the magnetic resonance imaging (MRI) and the anatomy itself for the extent of resection [58]. For this reason, AC also permits the surgeon to monitor and rely on the functionality of patient while the patient is awake, and thereby increase the extent of resection. Cortical mapping of these areas (through a set of direct electrodes that stimulate or inhibit cortical functions) remains the optimal option to delineate the relationship of tumor to eloquent cortex in order to avoid damage of tissue that can compromise language or movement skills in patients (Figure 2).

Advantages of AC include local sedation, better postoperative KPS status, lower length of hospitalization, and a decreased economic burden for hospital stay [55].

In a 2017 clinical report by Quinones et al., patients subjected to AC had more gross total resection, as well as exhibiting an improved postoperative functional status and reduced postoperative morbidities in comparison to patients subjected to general anesthesia (GA) for resection of gliomas located in eloquent areas (93.3% vs. 81.1% respectively) [54].

Interestingly, Quinones et al. also observed better resections in the AC group compared to the GA group (25.9% vs. 6.5%, respectively). Regarding the length of hospitalization, the statistics showed a three-day reduction in the AC group when compared with the GA group (4.2 days vs. 7.9 days, respectively) [54] (Table 1). This study thus highlights the various benefits of AC and suggested the power of this operative approach to replace traditional methods. Kim et al. recently evaluated the neurological status of 309 patients subjected to AC or standard procedures to treat tumors near or involving eloquent areas. The results demonstrated enhanced neurological status in the early postoperative state, and at three months after resection when safe margins of resection were determined through cortical mapping; 9% neurological deficits were seen with mapping vs. 21% neurological deficits without mapping [59]. Additionally, Lau et al. in 2017 assessed the accuracy of the surgical limits provided by AC, and found a 79.6% overall precision of intraoperative perception of gross total or subtotal resection achieved under AC. Interestingly, they also described higher quantitative perception accuracy when 1p19q co-deletion was absent (96.9% with negative vs. 81.5% with positive 1p19q co-deletion) [60]. Following the same premise, a clinical trial led by Bebawy et al. investigating the quality of recovery in the immediate postoperative state and 30 and 90 days after surgery with AC and asleep craniotomy. No data has been released yet; however, it is expected that the results will be consistent with previous findings supporting AC over general anesthesia [61].

Due to the complexity of AC, however, a multidisciplinary team composed of surgeons, anesthesiologists, and neurologists is required, as well as a meticulous selection and preparation process for patients undergoing surgery. Further evidence has demonstrated that AC is not a traumatic experience for the patients. A study developed by Khalid et al. in 2015, demonstrated that patients did not express any more discomfort than anticipated, reinforcing that AC is a well-tolerated procedure [62].

Confocal Intraoperative Microscopy: Near infrared (NIR) confocal endomicroscopy was first used to delineate infiltrative GBM in vivo as a preclinical model in rodents in 2011 [63]. The study determined that NIR wavelength with this microscopic technique joined with indocyanine green (ICG) detects fluorescence of tumor cells. Compared to NIR and ICG macroscopic imaging, NIR confocal endomicroscopy with ICG improved the sensitivity of tumor visualization, as it revealed not only discrete tumor cells and satellite peritumoral cells, but also subcellular and microvascular fluorescent structures in these regions. These findings elucidate the complex information that macroscopic and microscopic in vivo ICG imaging could offer with regard to brain tumor cell infiltration. Such structures have also been identified in clinical models in the past decade; the first report on confocal microscopy in neurosurgery 2010, known as neurolasermicroscopy (NLM), was used to assess cell density, mitotic features, necrosis, and microvascular cell proliferation of GBM; all of which highly facilitate tumor diagnosis during neurosurgery. Eschbacher et al. performed 50 microsurgeries of brain tumors including a hemangioblastoma, several grades of gliomas, meningiomas, and schwannomas, using confocal microscopy to visualize the regions in vivo with fluorescein sodium (FS) contrast. He also performed neuropathological traditional analysis of a subset of images, separately [64]. The identification of various cytoarchitectures of the different tumors from intraoperative confocal imaging was found to be consistent with the results from pathology, further demonstrating the potential of this powerful novel approach to diagnose brain tumors.

As with the previously described novel techniques, NIR technology and the more general microscopic techniques need to be further developed in order to expand the microscopic window with minimal interruption to the surgical workflow.

Fluorescence-Guided Surgery (FGS): The first application of an exogenous fluorescent marker used to resect brain tumors was performed by Moore et al. in 1948 [65]. Soon thereafter, several researchers supported the benefits of fluorescence for brain cancer surgical guidance, such as Stummer et al., 1997, who showed a 100% specificity and 85% sensitivity in brain tumor removal using 5-aminolevulinic acid-induced porphyrin fluorescence (5-ALA-PpIX) [66].

5-ALA is a photosensitive substance precursor of protoporphyrin IX (PpIX) (mainly visible with a wavelength of 375–475 nm) that turns into a red fluorescent emission signal after mitochondrial metabolization. 5-ALA is the only fluorophore that has been utilized in different clinical trials to identify tumor location, histopathological features, investigate pre- and post-operative MRI findings, and delineate eloquent areas in GBM surgery, with promising results augmenting patient survival. To date, 44 clinical studies have used 5-ALA for glioma surgery; these have demonstrated a GTR of 65% with 5-ALA compared to GTR of 35% for the group without the fluorophore. In addition, an increase in PFS of 3.8 months in the 5-ALA group was achieved (8.6 vs. 4.8 months, respectively) [67]. The real-time visualization of tumor presence or absence provided by this fluorophore offers immediate feedback to the surgeon who decides the delimitations of the tumor.

However, adverse effects after 5-ALA systemic administration have limited its application (nausea, mild hypotension, elevated liver enzymes, photosensitivity, and neurological deficits) [68,69]. Other common exogenous agents implemented in neurosurgery are ICG, FS which are described in Table 2.

Although the beneficial impact of FGS for tumor resection is evident, the burden of cost for its implementation, including equipment purchase, dose determination, and training of specific personnel must be considered. Additional evidence is necessary to consolidate the advantages obtained from this technology.

Laser Interstitial Thermal Therapy for GBM Ablation: Laser-interstitial thermal therapy (LITT), as a cancer treatment, is a thermocoagulative therapy in which laser irradiation is percutaneously delivered by an optical fiber into tissue which causes heat-generated necrosis of the tissue and hence, ablation of the targeted lesion [70]. Specifically, LITT coagulates tissue by selectively transmitting focal laser energy with interstitial hyperthermia and calculating a precise region of ablation based on the Arrhenius equation [71,72]. The delivery technique of laser therapy has thus allowed greater reachability to various tissue types or deeper-seated lesions.

LITT has been used to treat a wide range of brain tumors, such as resistant metastatic tumors [73,74], astrocytoma, chordoma, meningioma, and GBM [70]. Due to their aggressiveness, HGG are undoubtedly of keen interest for treatment with LITT as demonstrated by several studies. A multicenter study evaluated 34 consecutive difficult-to-access HGGs treated with LITT (under the NeuroBlate^®^ System, Monteris Medical Group, Plymouth, UK) at three different institutions in the US found a significant relationship between the cytoreductive effect of hyperthermia and PFS. Evidently, it has thus become useful to equivalently compare the surgical resection with the extent of ablation regarding patient overall survival [75]. Likewise, in their clinical review, Norred & Johnson compared the extension of survival of conventional resection techniques with LITT therapy, reinforcing the relatively short procedure and the amenability this therapy gives not to only multiple treatments, but also to inoperable or deep-seated tumors. Moreover, they also highlighted the potential adaptability LITT may have with nanotherapeutic models as a more advanced therapy, both diminishing the thermal damage to tissues and allowing for deeper optical penetration when considering the value of the cooling catheter [70]. Recurrent GBM treatment with LITT accompanied by MR thermal imaging (MRTI) has been recently explored in a 16-patient clinical report that compared overall survival of traditional chemotherapy to LITT. All patients had received TMZ and exhibited a median survival of 9.4 months; however, with LITT, their median survival increased to 11.2 months [73]. Carpentier et al. later showed the significant potential of LITT as one-day procedure salvage therapy in patients with recurrent GBM. They observed that the patients’ PFS had a mean/median of 37/30 days, whereas mean/median overall survival after LITT was 10.5/10 months [74].

The limitations of the small number of studies investigating LITT and HGGs should be carefully considered. As the evident heterogeneity of HGGs, and notably of GBMs, play a pivotal role in patient outcomes, the genetic characteristics of the patients’ tumors must be taken into account for survival analysis after LITT [74,75]. Overall, future investigations must find the optimal therapeutic regimen of LITT in controlled clinical trials.

Intraoperative Mass spectrometry (MS): is an analytical technique that identifies and characterizes molecules by taking into account their masses and fragmentation patterns, respectively, at the nanometer scale [76,77,78]. By using a matrix-assisted laser desorption/ionization technology, MS is an important approach for investigating the spatial arrangement of molecules in biological tissues in a sensitive and specific manner [78,79]. The desorption electrospray ionization (DESI) method, an extension of MS introduced in 2004 [80], allow the analysis of intact molecules and direct sampling of biological tissues [81].

**Figure 3:**
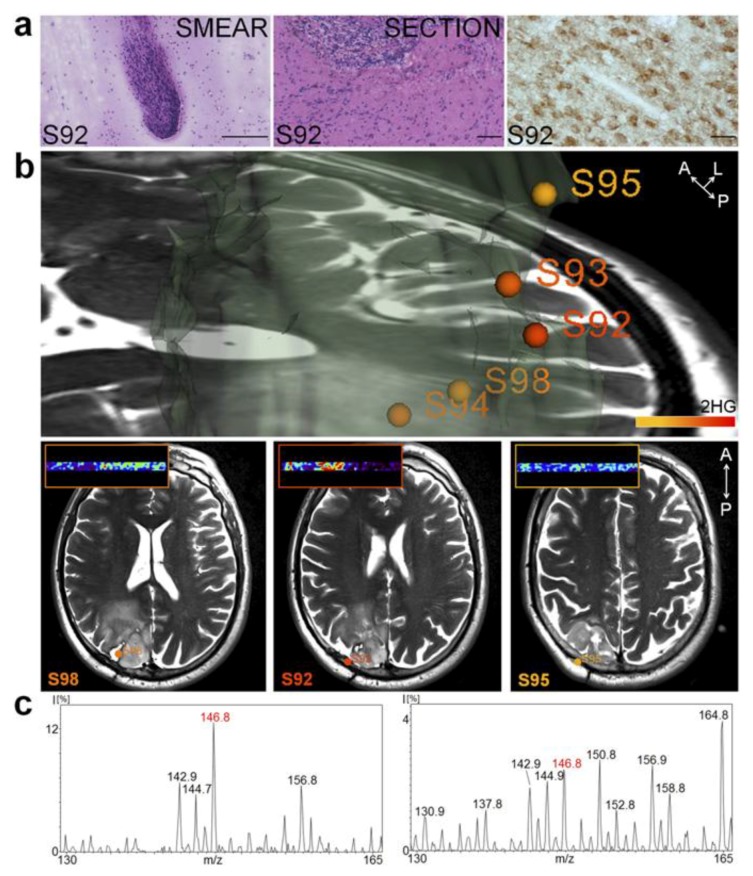
Intraoperative mass spectrometry of an onco-metabolite to guide brain tumor resection. (**a**) H & E-stained smear (**Left**), frozen tissue section (**Center**), and immunohistochemistry using an IDH1 R132H point mutation-specific antibody in an oligoastrocytoma grade III samples; (**b**) 3D tumor volume representation showing normalized 2-hydroxyglutarate (2-HG) signal represented with a warm color scale (lowest (yellow) to highest (red)); (**c**) Negative ion mode DESI mass spectra obtained from a smear (**Left**) and a section from an oligoastrocytoma grade III (**Right**). Used with permission from Santagata et al. [82].

By integrating MS in the operating room, surgeons can use it as a real-time guide to delineate tumors by acquiring complex molecular information in real-time. Intraoperative examination of brain tumors has since been increasingly employed. Agar et al. have presented a study in which they combined DESI into stereotactic surgery in order to develop a resection technique that took advantage of the speed and high sensitivity of MS. They fully integrated this methodology with the surgical procedure, accompanying it with intraoperative MRI and positron-emission tomography (PET) scans. Moreover, this MS approach does not require molecular agents or systemic injections [76]. Santagata et al. further capitalized on the time-saving approach that MS provides when they showed the efficiency of detecting validated onco-metabolites, such as the molecular marker generated from isocitrate dehydrogenase 1 mutant gliomas (2-hydroxyglutarate), by using ambient MS without any necessary sample preparation [82]. This validation immediately provided critical diagnostic, prognostic, and predictive information that would otherwise depend on post-operative pathological evaluations (Figure 3).

This approach thus highlights the advantage MS offers by enabling metabolite-imaging during brain surgery, notably one found in not only most of secondary GBMs but also 80% of grade II and grade III [82,83]. A 2017 study also used DESI-MS to rapidly analyze tissues during glioma resection and consistently indicate the reliability, simplicity, and integrative features of DESI-MS. Preliminary data from ten patients also demonstrated an overall 93% sensitivity and 83% specificity of surgical demarcation via an estimation of high tumor cell percentage given by DESI-MS [84]. Furthermore, these results uphold the significance of MS for maximal safe tumor resection and hence improved patient survival.

To date, MS has largely been explored due to an increasing demand of sensitive techniques to treat challenging tumors such as HGGs. Yet, this novel technology remains in its clinical infancy, as the molecular findings it presents remain limited.

## 5. Optical Coherence Tomography (OCT): Future Strategy for Glioma Surgery

In neurosurgery, the need for a non-invasive approach that allows real-time identification of cancer and non-cancer tissue is still open. Intraoperative examination of resected cancer tissues without processing of the sample (fixation or freezing) is an advantage of optical coherence tomography. Strong evidence from preclinical experiments supports its applicability for brain cancer.

**Figure 4:**
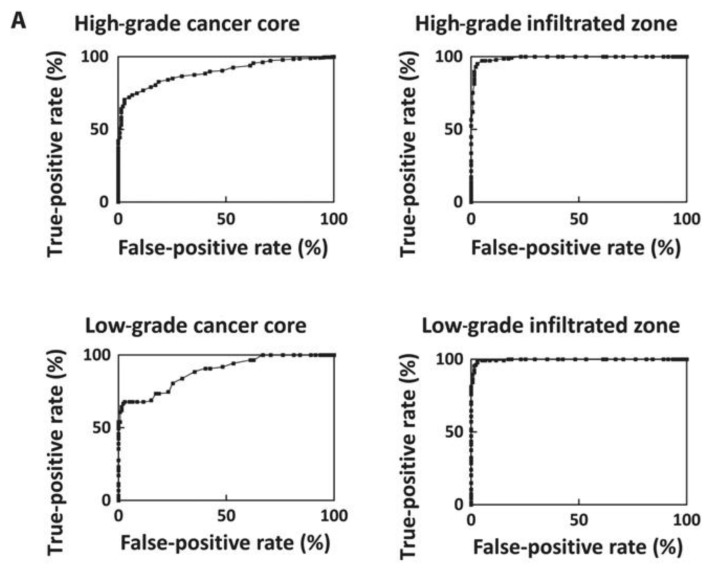

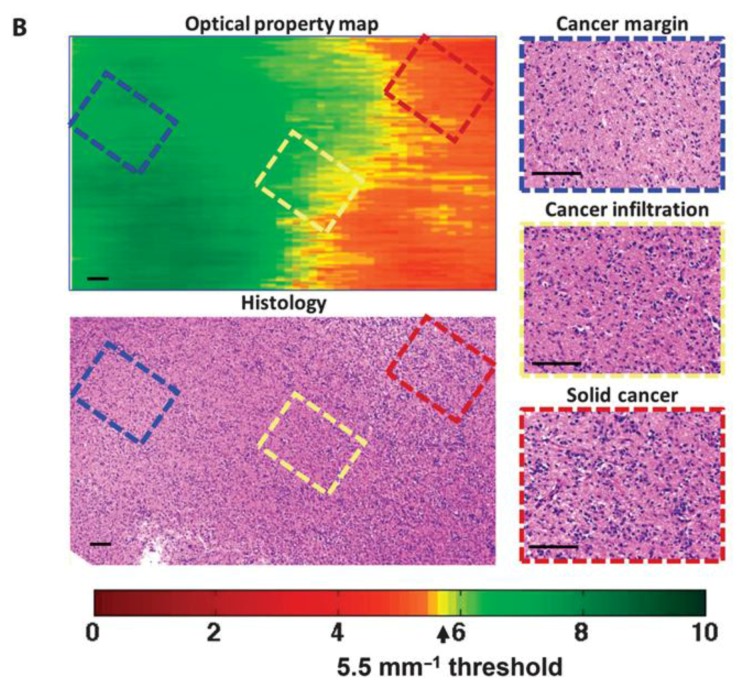
OCT for tumor tissue identification in ex vivo samples. (**A**) Sensitivity and specificity rates for cancer core and infiltrated zone in tissues obtained from a set of 16 samples; (**B**) OCT attenuation map results in cancer core and infiltrated zones, and its correlation with histology. Used with permission from Kut et al. [85].

OCT is a non-invasive label-free technique based on the interaction of light emission and tissue that illustrates two- and three-dimensional acquisitions of images in a color-coded map [85]. With OCT, it is possible to capture higher-resolution images from deeper structures in tissues (up to 2 mm, approximately). It has been applied in a broad spectrum of pathologies in organs such as the brain, breast, and stomach [85] (Figure 4).

For brain cancer specifically, in an ex vivo model, OCT provided high-quality two-dimensional imaging in non-cancer and cancer tissues, with an optical attenuation values of 9% vs. 33% in non-cancer and cancer samples, respectively [85]. Using a different set of glioma samples, the researchers found that OCT showed a specificity of 50–70% and a sensitivity of 40–100% when differentiating HGG and LGG, with a correlation between histopathology and the color-coding map among samples [85].

Besides illustrating tumor hypercellularity, OCT also identified necrotic areas shown as hypo intense signals in the map. Tumor angiogenesis, as well as brain blood flow [86] and blood brain barrier (BBB) responses across a variety of sites, can also be studied with OCT, as demonstrated in animal models of von Hippel-Lindau [87]. Further analysis of this technology is much needed in order to determine whether tumor visualization is enhanced when OCT is used in comparison to traditional methods.

## 6. Conclusions

Glioblastoma is a disease that cannot be treated by surgery alone. Despite its inevitable recurrence, tumor excision is still an essential part to extend patients’ survival. Technological advances that allow real-time and quantitative visualization of cancer-infiltration and non-cancer parenchyma have become essential for brain tumor surgery. The aforementioned surgical procedures have shown efficacy in achieving safe maximal resection while preserving the patient’s functionality, and facilitating decision-making in the operating room. Still, the remaining technological challenges call for scientific efforts towards a better understanding of brain tumor initiation and development, ultimately leading to a solution for treating this devastating disease.

## Figures and Tables

**Table 1:** Achieved resection of patients undergoing awake craniotomy (AC) for resection gliomas located in eloquent regions.

Percentage of Resection	Type of Surgery		*p* Value
	Asleep (*n* = 31)	Awake (*n* = 27)	
100%	2 (6.5%)	7 (25.9%)	0.041
≥95%, <100%	11 (35.5%)	10 (37.0%)	0.902
<95%	18 (58.1%)	10 (37.0%)	0.110

Boldface values indicate significance *p* < 0.05. (Reproduced from: Eseonu et al. [54]).

**Table 2:** Extrinsic fluorescent contrast agents.

Name	Specifications
ICG	FDA approved for injection in cardiology and ophthalmology Used to determine tumor angiogenesis or vascular malformations (i.e., Hemangioblastoma) [59] Emission peak of 820 nm Depth approximately of >350 μm [55] AE: anaphylactic reactions
Hypericin	A red-colored fluorophore with a sensitivity of 94% and a specificity of 100% for glioma surgery [60] Emits signal at 590 nm AE: anaphylactic reactions
FS	Green fluorophore, used in glioma surgery to improve gross total resection (30–80% of tumor removal) Excited at 480 nm AE: anaphylactic reactions and yellow pigmentation of skin, mucosa and urine [54,61,62,63].

FDA: Food and Drug Administration, ICG: indocyanine green, FS: fluorescein sodium, AE: adverse effects. (Modified from Senders et al. [67]).

## References

[1] Lacroix M., Abi-Said D., Fourney D.R., Gokaslan Z.L., Shi W., DeMonte F., Lang F.F., McCutcheon I.E., Hassenbusch S.J., Holland E. (2001). A multivariate analysis of 416 patients with glioblastoma multiforme: Prognosis, extent of resection, and survival. J. Neurosurg..

[2] Laws E.R., Parney I.F., Huang W., Anderson F., Morris A.M., Asher A., Lillehei K.O., Bernstein M., Brem H., Sloan A. (2003). Survival following surgery and prognostic factors for recently diagnosed malignant glioma: Data from the glioma outcomes project. J. Neurosurg..

[3] Sanai N., Polley M.Y., McDermott M.W., Parsa A.T., Berger M.S. (2011). An extent of resection threshold for newly diagnosed glioblastomas. J. Neurosurg..

[4] de Robles P., Fiest K.M., Frolkis A.D., Pringsheim T., Atta C., St Germaine-Smith C., Day L., Lam D., Jette N. (2015). The worldwide incidence and prevalence of primary brain tumors: A systematic review and meta-analysis. Neuro Oncol..

[5] Stupp R., Mason W.P., van den Bent M.J., Weller M., Fisher B., Taphoorn M.J., Belanger K., Brandes A.A., Marosi C., Bogdahn U. (2005). Radiotherapy plus concomitant and adjuvant temozolomide for glioblastoma. N. Engl. J. Med..

[6] Markert J.M. (2012). The role of early resection vs. biopsy in the management of low-grade gliomas. JAMA.

[7] Chaichana K.L., Jusue-Torres I., Navarro-Ramirez R., Raza S.M., Pascual-Gallego M., Ibrahim A., Hernandez-Hermann M., Gomez L., Ye X., Weingart J.D. (2014). Establishing percent resection and residual volume thresholds affecting survival and recurrence for patients with newly diagnosed intracranial glioblastoma. Neuro Oncol..

[8] Chaichana K.L., Jusue-Torres I., Lemos A.M., Gokaslan A., Cabrera-Aldana E.E., Ashary A., Olivi A., Quinones-Hinojosa A. (2014). The butterfly effect on glioblastoma: Is volumetric extent of resection more effective than biopsy for these tumors?. J. Neurooncol..

[9] Senft C., Bink A., Franz K., Vatter H., Gasser T., Seifert V. (2011). Intraoperative mri guidance and extent of resection in glioma surgery: A randomised, controlled trial. Lancet Oncol..

[10] Tilghman J., Wu H., Sang Y., Shi X., Guerrero-Cazares H., Quinones-Hinojosa A., Eberhart C.G., Laterra J., Ying M. (2014). Hmmr maintains the stemness and tumorigenicity of glioblastoma stem-like cells. Cancer Res..

[11] Huang Z., Cheng L., Guryanova O.A., Wu Q., Bao S. (2010). Cancer stem cells in glioblastoma-molecular signaling and therapeutic targeting. Protein Cell.

[12] Iacob G., Dinca E.B. (2009). Current data and strategy in glioblastoma multiforme. J. Med. Life.

[13] Lara-Velazquez M., Akinduro O.O., Reimer R., Woodmansee W.W., Quinones-Hinojosa A. (2017). Stem cell therapy and its potential role in pituitary disorders. Curr. Opin. Endocrinol. Diabetes Obes..

[14] Lathia J.D., Liu H. (2017). Overview of cancer stem cells and stemness for community oncologists. Target. Oncol..

[15] Amelot A., De Cremoux P., Quillien V., Polivka M., Adle-Biassette H., Lehmann-Che J., Françoise L., Carpentier A.F., George B., Mandonnet E. (2015). Idh-mutation is a weak predictor of long-term survival in glioblastoma patients. PLoS ONE.

[16] Verhaak R.G., Hoadley K.A., Purdom E., Wang V., Qi Y., Wilkerson M.D., Miller C.R., Ding L., Golub T., Mesirov J.P. (2010). Integrated genomic analysis identifies clinically relevant subtypes of glioblastoma characterized by abnormalities in PDGFRA, IDH1, EGFR, and NF1. Cancer Cell.

[17] Oh Y.T., Cho H.J., Kim J., Lee J.H., Rho K., Seo Y.J., Choi Y.S., Jung H.J., Song H.S., Kong D.S. (2014). Translational validation of personalized treatment strategy based on genetic characteristics of glioblastoma. PLoS ONE.

[18] Phillips H.S., Kharbanda S., Chen R., Forrest W.F., Soriano R.H., Wu T.D., Misra A., Nigro J.M., Colman H., Soroceanu L. (2006). Molecular subclasses of high-grade glioma predict prognosis, delineate a pattern of disease progression, and resemble stages in neurogenesis. Cancer Cell.

[19] Theeler B.J., Gilbert M.R. (2015). Advances in the treatment of newly diagnosed glioblastoma. BMC Med..

[20] Ostrom Q.T., Gittleman H., Liao P., Rouse C., Chen Y., Dowling J., Wolinsky Y., Kruchko C., Barnholtz-Sloan J. (2014). Cbtrus statistical report: Primary brain and central nervous system tumors diagnosed in the united states in 2007–2011. Neuro Oncol..

[21] Rivera A.L., Pelloski C.E., Gilbert M.R., Colman H., De La Cruz C., Sulman E.P., Bekele B.N., Aldape K.D. (2010). Mgmt promoter methylation is predictive of response to radiotherapy and prognostic in the absence of adjuvant alkylating chemotherapy for glioblastoma. Neuro Oncol..

[22] Bhandari M., Gandhi A.K., Devnani B., Kumar P., Sharma D.N., Julka P.K. (2017). Comparative study of adjuvant temozolomide six cycles versus extended 12 cycles in newly diagnosed glioblastoma multiforme. J. Clin. Diagn. Res..

[23] Olar A., Aldape K.D. (2014). Using the molecular classification of glioblastoma to inform personalized treatment. J. Pathol..

[24] Gaspar L.E., Fisher B.J., Macdonald D.R., LeBer D.V., Halperin E.C., Schold S.C., Cairncross J.G. (1992). Supratentorial malignant glioma: Patterns of recurrence and implications for external beam local treatment. Int. J. Radiat. Oncol. Biol. Phys..

[25] Wong E.T., Hess K.R., Gleason M.J., Jaeckle K.A., Kyritsis A.P., Prados M.D., Levin V.A., Yung W.K. (1999). Outcomes and prognostic factors in recurrent glioma patients enrolled onto phase ii clinical trials. J. Clin. Oncol..

[26] Chaichana K.L., Zadnik P., Weingart J.D., Olivi A., Gallia G.L., Blakeley J., Lim M., Brem H., Quinones-Hinojosa A. (2013). Multiple resections for patients with glioblastoma: Prolonging survival. J. Neurosurg..

[27] Roy S., Lahiri D., Maji T., Biswas J. (2015). Recurrent glioblastoma: Where we stand. South Asian J. Cancer.

[28] Stockelmaier L., Renovanz M., Konig J., Nickel K., Hickmann A.K., Mayer-Steinacker R., Nadji-Ohl M., Ganslandt O., Bullinger L., Wirtz C.R. (2017). Therapy for recurrent high-grade gliomas: Results of a prospective multicenter study on health-related quality of life. World Neurosurg..

[29] Rahmathulla G., Hovey E.J., Hashemi-Sadraei N., Ahluwalia M.S. (2013). Bevacizumab in high-grade gliomas: A review of its uses, toxicity assessment, and future treatment challenges. OncoTargets Ther..

[30] Vredenburgh J.J., Desjardins A., Herndon J.E., Dowell J.M., Reardon D.A., Quinn J.A., Rich J.N., Sathornsumetee S., Gururangan S., Wagner M. (2007). Phase ii trial of bevacizumab and irinotecan in recurrent malignant glioma. Clin. Cancer Res..

[31] Friedman H.S., Prados M.D., Wen P.Y., Mikkelsen T., Schiff D., Abrey L.E., Yung W.K., Paleologos N., Nicholas M.K., Jensen R. (2009). Bevacizumab alone and in combination with irinotecan in recurrent glioblastoma. J. Clin. Oncol..

[32] Attenello F.J., Mukherjee D., Datoo G., McGirt M.J., Bohan E., Weingart J.D., Olivi A., Quinones-Hinojosa A., Brem H. (2008). Use of gliadel (BCNU) wafer in the surgical treatment of malignant glioma: A 10-year institutional experience. Ann. Surg. Oncol..

[33] Chaichana K.L., Kone L., Bettegowda C., Weingart J.D., Olivi A., Lim M., Quinones-Hinojosa A., Gallia G.L., Brem H. (2015). Risk of surgical site infection in 401 consecutive patients with glioblastoma with and without carmustine wafer implantation. Neurol. Res..

[34] Chaichana K.L., Zaidi H., Pendleton C., McGirt M.J., Grossman R., Weingart J.D., Olivi A., Quinones-Hinojosa A., Brem H. (2011). The efficacy of carmustine wafers for older patients with glioblastoma multiforme: Prolonging survival. Neurol. Res..

[35] Grossman R., Burger P., Soudry E., Tyler B., Chaichana K.L., Weingart J., Olivi A., Gallia G.L., Sidransky D., Quinones-Hinojosa A. (2015). Mgmt inactivation and clinical response in newly diagnosed gbm patients treated with gliadel. J. Clin. Neurosci..

[36] McGirt M.J., Than K.D., Weingart J.D., Chaichana K.L., Attenello F.J., Olivi A., Laterra J., Kleinberg L.R., Grossman S.A., Brem H. (2009). Gliadel (BCNU) wafer plus concomitant temozolomide therapy after primary resection of glioblastoma multiforme. J. Neurosurg..

[37] Kaloshi G., Diamandi P., Cakani B., Brace G., Rroji A., Petrela M. (2015). The added value of bevacizumab concomitantly administered with carboplatin versus carboplatin alone in patients with recurrent glioblastomas. Tumori.

[38] Elizabeth G. (2009). Cediranib Maleate and Cilengitide in Treating Patients with Progressive or Recurrent Glioblastoma. https://clinicaltrials.gov/ct2/show/NCT00979862.

[39] Vogelbaum M.A. (2002). Erlotinib in Treating Patients with Recurrent or Progressive Glioblastoma Multiforme. https://clinicaltrials.gov/ct2/show/study/NCT00979862.

[40] Rich J.N., Reardon D.A., Peery T., Dowell J.M., Quinn J.A., Penne K.L., Wikstrand C.J., Duyn L.B.V., Dancey J.E., McLendon R.E. (2004). Phase ii trial of gefitinib in recurrent glioblastoma. J. Clin. Oncol..

[41] Gil-Gil M.J., Mesia C., Rey M., Bruna J. (2013). Bevacizumab for the treatment of glioblastoma. Clin. Med. Insights Oncol..

[42] Davies A.M., Weinberg U., Palti Y. (2013). Tumor treating fields: A new frontier in cancer therapy. Ann. N. Y. Acad. Sci..

[43] Stupp R., Taillibert S., Kanner A.A., Kesari S., Steinberg D.M., Toms S.A., Taylor L.P., Lieberman F., Silvani A., Fink K.L. (2015). Maintenance therapy with tumor-treating fields plus temozolomide vs. temozolomide alone for glioblastoma: A randomized clinical trial. JAMA.

[44] US Cancer Statistics Working Group (2017). United States Cancer Statistics: 1999–2014 Incidence and Mortality Web-Based Report.

[45] Bloch O., Han S.J., Cha S., Sun M.Z., Aghi M.K., McDermott M.W., Berger M.S., Parsa A.T. (2012). Impact of extent of resection for recurrent glioblastoma on overall survival: Clinical article. J. Neurosurg..

[46] Eseonu C.I., ReFaey K., Garcia O., Raghuraman G., Quinones-Hinojosa A. (2017). Volumetric analysis of extent of resection, survival, and surgical outcomes for insular gliomas. World Neurosurg..

[47] Eseonu C.I., Eguia F., ReFaey K., Garcia O., Rodriguez F.J., Chaichana K., Quinones-Hinojosa A. (2017). Comparative volumetric analysis of the extent of resection of molecularly and histologically distinct low grade gliomas and its role on survival. J. Neurooncol..

[48] McGirt M.J., Chaichana K.L., Gathinji M., Attenello F.J., Than K., Olivi A., Weingart J.D., Brem H., Quinones-Hinojosa A.R. (2009). Independent association of extent of resection with survival in patients with malignant brain astrocytoma. J. Neurosurg..

[49] Brown T.J., Brennan M.C., Li M., Church E.W., Brandmeir N.J., Rakszawski K.L., Patel A.S., Rizk E.B., Suki D., Sawaya R. (2016). Association of the extent of resection with survival in glioblastoma: A systematic review and meta-analysis. JAMA Oncol..

[50] Hervey-Jumper S.L., Berger M.S. (2014). Role of surgical resection in low- and high-grade gliomas. Curr. Treat. Opt. Neurol..

[51] Reyns N., Leroy H.A., Delmaire C., Derre B., Le-Rhun E., Lejeune J.P. (2017). Intraoperative mri for the management of brain lesions adjacent to eloquent areas. Neurochirurgie.

[52] Ronkainen J., Tervonen O. (2006). Cost analysis of an open low-field (0.23t) mri unit: Effect of procedure shares in combined imaging, interventional, and neurosurgical use. Acta Radiol..

[53] Eseonu C.I., ReFaey K., Garcia O., John A., Quinones-Hinojosa A., Tripathi P. (2017). Awake craniotomy anesthesia: A comparison of the monitored anesthesia care and asleep-awake-asleep techniques. World Neurosurg..

[54] Eseonu C.I., Rincon-Torroella J., ReFaey K., Lee Y.M., Nangiana J., Vivas-Buitrago T., Quinones-Hinojosa A. (2017). Awake craniotomy vs. craniotomy under general anesthesia for perirolandic gliomas: Evaluating perioperative complications and extent of resection. Neurosurgery.

[55] Eseonu C.I., Rincon-Torroella J., ReFaey K., Quinones-Hinojosa A. (2017). The cost of brain surgery: Awake vs. asleep craniotomy for perirolandic region tumors. Neurosurgery.

[56] Quinones-Hinojosa A., Ojemann S.G., Sanai N., Dillon W.P., Berger M.S. (2003). Preoperative correlation of intraoperative cortical mapping with magnetic resonance imaging landmarks to predict localization of the broca area. J. Neurosurg..

[57] Walker J.A., Quinones-Hinojosa A., Berger M.S. (2004). Intraoperative speech mapping in 17 bilingual patients undergoing resection of a mass lesion. Neurosurgery.

[58] Southwell D.G., Hervey-Jumper S.L., Perry D.W., Berger M.S. (2016). Intraoperative mapping during repeat awake craniotomy reveals the functional plasticity of adult cortex. J. Neurosurg..

[59] Kim S.S., McCutcheon I.E., Suki D., Weinberg J.S., Sawaya R., Lang F.F., Ferson D., Heimberger A.B., DeMonte F., Prabhu S.S. (2009). Awake craniotomy for brain tumors near eloquent cortex: Correlation of intraoperative cortical mapping with neurological outcomes in 309 consecutive patients. Neurosurgery.

[60] Lau D., Hervey-Jumper S.L., Han S.J., Berger M.S. (2017). Intraoperative perception and estimates on extent of resection during awake glioma surgery: Overcoming the learning curve. J. Neurosurg..

[61] Bebawy J. (2014). Quality of Recovery Awake versus Asleep Craniotomy. https://clinicaltrials.gov/ct2/show/NCT02228993.

[62] Bajunaid K.M., Ajlan A.M. (2015). Awake craniotomy. A patient’s perspective. Neurosciences.

[63] Martirosyan N.L., Cavalcanti D.D., Eschbacher J.M., Delaney P.M., Scheck A.C., Abdelwahab M.G., Nakaji P., Spetzler R.F., Preul M.C. (2011). Use of in vivo near-infrared laser confocal endomicroscopy with indocyanine green to detect the boundary of infiltrative tumor. J. Neurosurg..

[64] Eschbacher J., Martirosyan N.L., Nakaji P., Sanai N., Preul M.C., Smith K.A., Coons S.W., Spetzler R.F. (2012). In vivo intraoperative confocal microscopy for real-time histopathological imaging of brain tumors. J. Neurosurg..

[65] Moore G.E., Peyton W.T., French L.A., Walker W.W. (1948). The clinical use of fluorescein in neurosurgery; the localization of brain tumors. J. Neurosurg..

[66] Murray K.J. (1982). Improved surgical resection of human brain tumors: Part I. A preliminary study. Surg. Neurol..

[67] Senders J.T., Muskens I.S., Schnoor R., Karhade A.V., Cote D.J., Smith T.R., Broekman M.L. (2017). Agents for fluorescence-guided glioma surgery: A systematic review of preclinical and clinical results. Acta Neurochir..

[68] Nguyen Q.T., Tsien R.Y. (2013). Fluorescence-guided surgery with live molecular navigation—A new cutting edge. Nat. Rev. Cancer.

[69] Mansouri A., Mansouri S., Hachem L.D., Klironomos G., Vogelbaum M.A., Bernstein M., Zadeh G. (2016). The role of 5-aminolevulinic acid in enhancing surgery for high-grade glioma, its current boundaries, and future perspectives: A systematic review. Cancer.

[70] Norred S.E., Johnson J.A. (2014). Magnetic resonance-guided laser induced thermal therapy for glioblastoma multiforme: A review. Biomed. Res. Int..

[71] Rahmathulla G., Recinos P.F., Valerio J.E., Chao S., Barnett G.H. (2012). Laser interstitial thermal therapy for focal cerebral radiation necrosis: A case report and literature review. Stereotact. Funct. Neurosurg..

[72] Laidler K.J. (1984). The development of the arrhenius equation. J. Chem. Educ..

[73] Schwarzmaier H.J., Eickmeyer F., von Tempelhoff W., Fiedler V.U., Niehoff H., Ulrich S.D., Yang Q., Ulrich F. (2006). Mr-guided laser-induced interstitial thermotherapy of recurrent glioblastoma multiforme: Preliminary results in 16 patients. Eur. J. Radiol..

[74] Carpentier A., Chauvet D., Reina V., Beccaria K., Leclerq D., McNichols R.J., Gowda A., Cornu P., Delattre J.Y. (2012). Mr-guided laser-induced thermal therapy (LITT) for recurrent glioblastomas. Lasers Surg. Med..

[75] Mohammadi A.M., Hawasli A.H., Rodriguez A., Schroeder J.L., Laxton A.W., Elson P., Tatter S.B., Barnett G.H., Leuthardt E.C. (2014). The role of laser interstitial thermal therapy in enhancing progression-free survival of difficult-to-access high-grade gliomas: A multicenter study. Cancer Med..

[76] Agar N.Y., Golby A.J., Ligon K.L., Norton I., Mohan V., Wiseman J.M., Tannenbaum A., Jolesz F.A. (2011). Development of stereotactic mass spectrometry for brain tumor surgery. Neurosurgery.

[77] Pacholski M.L., Winograd N. (1999). Imaging with mass spectrometry. Chem. Rev..

[78] Stoeckli M., Chaurand P., Hallahan D.E., Caprioli R.M. (2001). Imaging mass spectrometry: A new technology for the analysis of protein expression in mammalian tissues. Nat. Med..

[79] Karas M., Bachmann D., Bahr U.E., Hillenkamp F. (1987). Matrix-assisted ultraviolet laser desorption of non-volatile compounds. Int. J. Mass Spectrom. Ion Process..

[80] Takats Z., Wiseman J.M., Gologan B., Cooks R.G. (2004). Mass spectrometry sampling under ambient conditions with desorption electrospray ionization. Science.

[81] Young R.M., Jamshidi A., Davis G., Sherman J.H. (2015). Current trends in the surgical management and treatment of adult glioblastoma. Ann. Transl. Med..

[82] Santagata S., Eberlin L.S., Norton I., Calligaris D., Feldman D.R., Ide J.L., Liu X., Wiley J.S., Vestal M.L., Ramkissoon S.H. (2014). Intraoperative mass spectrometry mapping of an onco-metabolite to guide brain tumor surgery. Proc. Natl. Acad. Sci. USA.

[83] Yan H., Parsons D.W., Jin G., McLendon R., Rasheed B.A., Yuan W., Kos I., Batinic-Haberle I., Jones S., Riggins G.J. (2009). IDH1 and IDH2 mutations in gliomas. N. Engl. J. Med..

[84] Pirro V., Alfaro C.M., Jarmusch A.K., Hattab E.M., Cohen-Gadol A.A., Cooks R.G. (2017). Intraoperative assessment of tumor margins during glioma resection by desorption electrospray ionization-mass spectrometry. Proc. Natl. Acad. Sci. USA.

[85] Kut C., Chaichana K.L., Xi J., Raza S.M., Ye X., McVeigh E.R., Rodriguez F.J., Quinones-Hinojosa A., Li X. (2015). Detection of human brain cancer infiltration ex vivo and in vivo using quantitative optical coherence tomography. Sci. Transl. Med..

[86] Iftimia N., Hammer D.X., Mujat M., Desphande V., Cizginer S., Brugge W. (2009). Optical coherence tomography imaging for cancer diagnosis and therapy guidance. Conf. Proc. IEEE Eng. Med. Biol. Soc..

[87] Sufan R.I., Moriyama E.H., Mariampillai A., Roche O., Evans A.J., Alajez N.M., Vitkin I.A., Yang V.X., Liu F.F., Wilson B.C. (2009). Oxygen-independent degradation of HIF-alpha via bioengineered VHL tumour suppressor complex. EMBO Mol. Med..

